# Reducing US cardiovascular disease burden and disparities through national and targeted dietary policies: A modelling study

**DOI:** 10.1371/journal.pmed.1002311

**Published:** 2017-06-06

**Authors:** Jonathan Pearson-Stuttard, Piotr Bandosz, Colin D. Rehm, Jose Penalvo, Laurie Whitsel, Tom Gaziano, Zach Conrad, Parke Wilde, Renata Micha, Ffion Lloyd-Williams, Simon Capewell, Dariush Mozaffarian, Martin O’Flaherty

**Affiliations:** 1 Department of Public Health and Policy, University of Liverpool, Liverpool, United Kingdom; 2 School of Public Health, Imperial College London, London, United Kingdom; 3 Department of Preventive Medicine and Education, Medical University of Gdańsk, Gdańsk, Poland; 4 Office of Community and Population Health, Montefiore Medical Center, New York, New York, United States of America; 5 Friedman School of Nutrition Science and Policy, Tufts University, Boston, Massachusetts, United States of America; 6 American Heart Association, Washington, District of Columbia, United States of America; 7 Division of Cardiovascular Medicine, Brigham and Women’s Hospital, Boston, Massachusetts, United States of America; Stanford University, UNITED STATES

## Abstract

**Background:**

Large socio-economic disparities exist in US dietary habits and cardiovascular disease (CVD) mortality. While economic incentives have demonstrated success in improving dietary choices, the quantitative impact of different dietary policies on CVD disparities is not well established. We aimed to quantify and compare the potential effects on total CVD mortality and disparities of specific dietary policies to increase fruit and vegetable (F&V) consumption and reduce sugar-sweetened beverage (SSB) consumption in the US.

**Methods and findings:**

Using the US IMPACT Food Policy Model and probabilistic sensitivity analyses, we estimated and compared the reductions in CVD mortality and socio-economic disparities in the US population potentially achievable from 2015 to 2030 with specific dietary policy scenarios: (a) a national mass media campaign (MMC) aimed to increase consumption of F&Vs and reduce consumption of SSBs, (b) a national fiscal policy to tax SSBs to increase prices by 10%, (c) a national fiscal policy to subsidise F&Vs to reduce prices by 10%, and (d) a targeted policy to subsidise F&Vs to reduce prices by 30% among Supplemental Nutrition Assistance Program (SNAP) participants only. We also evaluated a combined policy approach, combining all of the above policies. Data sources included the Surveillance, Epidemiology, and End Results Program, National Vital Statistics System, National Health and Nutrition Examination Survey, and published meta-analyses.

Among the individual policy scenarios, a national 10% F&V subsidy was projected to be most beneficial, potentially resulting in approximately 150,500 (95% uncertainty interval [UI] 141,400–158,500) CVD deaths prevented or postponed (DPPs) by 2030 in the US. This far exceeds the approximately 35,100 (95% UI 31,700–37,500) DPPs potentially attributable to a 30% F&V subsidy targeting SNAP participants, the approximately 25,800 (95% UI 24,300–28,500) DPPs for a 1-y MMC, or the approximately 31,000 (95% UI 26,800–35,300) DPPs for a 10% SSB tax.

Neither the MMC nor the individual national economic policies would significantly reduce CVD socio-economic disparities. However, the SNAP-targeted intervention might potentially reduce CVD disparities between SNAP participants and SNAP-ineligible individuals, by approximately 8% (10 DPPs per 100,000 population). The combined policy approach might save more lives than any single policy studied (approximately 230,000 DPPs by 2030) while also significantly reducing disparities, by approximately 6% (7 DPPs per 100,000 population).

Limitations include our effect estimates in the model; these estimates use interventional and prospective observational studies (not exclusively randomised controlled trials). They are thus imperfect and should be interpreted as the best available evidence. Another key limitation is that we considered only CVD outcomes; the policies we explored would undoubtedly have additional beneficial effects upon other diseases. Further, we did not model or compare the cost-effectiveness of each proposed policy.

**Conclusions:**

Fiscal strategies targeting diet might substantially reduce CVD burdens. A national 10% F&V subsidy would save by far the most lives, while a 30% F&V subsidy targeting SNAP participants would most reduce socio-economic disparities. A combined policy would have the greatest overall impact on both mortality and socio-economic disparities.

## Abstract 2

In a modeling study, Jonathan Pearson-Stuttard and colleagues estimate the potential impact of targeted dietary policies on cardiovascular disease burden in the US.

## Author summary

**Why was this study done?:**

- Suboptimal diet is a leading cause of cardiovascular disease, death, and health disparities. Dietary policies have the potential to reduce this burden.
- However, the potential benefits of policies targeting fruit, vegetable, and sugar-sweetened beverage consumption in the whole US population and among those participating in the Supplemental Nutrition Assistance Program (SNAP) have not been quantified.

**What did the researchers do and find?:**

- We modelled and compared the potential benefits of several dietary policies targeting fruit, vegetable, and sugar-sweetened beverage consumption.
- We found that a modest universal reduction in fruit and vegetable prices (10% subsidy) was most likely to reduce cardiovascular disease mortality, whilst a 30% fruit and vegetable subsidy offered to SNAP participants appeared most promising for reducing disparities in cardiovascular disease mortality.
- Finally, we found that a combination of all these policies potentially offered the biggest benefits in terms of reducing cardiovascular disease burden and also reducing disparities.

**What do these findings mean?:**

- Our findings highlight the potentially powerful effects of fiscal measures targeting diet in the US. Dietary policies could potentially reduce cardiovascular disease, death, and associated disparities.
- A modest subsidy of fruits and vegetables for all, accompanied by a larger subsidy for SNAP participants, might be most beneficial in terms of reducing the disease burden and disparities.

## Introduction

Cardiovascular disease (CVD) is declining in the US [1–3]. However, CVD remains the leading cause of mortality, generating approximately 800,000 deaths and 6 million hospital admissions annually [2]. Crucially, these burdens are highly unequal across the population, in particular according to socio-economic status (SES) [4].

Among modifiable risk factors [5], insufficient consumption of fruits and vegetables (F&Vs) [6–8] and excess intake of sugar-sweetened beverages (SSBs) [9] are important contributors to CVD. Furthermore, dietary patterns and intakes of these foods are worse among low-SES groups [10], making them important dietary targets for policy makers wishing to reduce CVD and also decrease disparities [11]. Policies to reduce F&V prices and increase SSB prices are effective measures for altering consumption, and may be especially effective among individuals with lower SES [12], who have worse CVD health [13]. However, the quantitative impact of different dietary policies on CVD mortality and socio-economic disparities is not well established.

To address these gaps, we quantified and compared the potential effects on total CVD mortality and CVD socio-economic disparities of specific dietary policies to increase F&V consumption and/or decrease SSB consumption in the US population up to 2030. Using empirical estimates of policy and food consumption effects, we evaluated both national policies and targeted policies for the Supplemental Nutrition Assistance Program (SNAP), the largest federal feeding programme, which serves approximately 46 million low-income Americans. For comparison, we also evaluated the potential impact of a national mass media campaign (MMC) on CVD deaths and socio-economic disparities.

## Methods

We modelled the potential effects of specific US dietary policies targeting F&Vs and SSBs from 2015 to 2030 using the previously validated US IMPACT Food Policy Model. We quantified and compared the associated change in dietary intake for a national MMC, national fiscal policies targeting F&Vs or SSBs, and a F&V targeted policy in SNAP participants, all by age, sex, and SNAP participation status. We also evaluated the joint associated change in dietary intake of combining these policies. We modelled the comparative effects upon coronary heart disease (CHD), stroke, and total CVD mortality, as well as the effect on CVD socio-economic disparities, comparing SNAP participants and SNAP-ineligible individuals.

### Data sources

The US population was estimated using data from the Surveillance, Epidemiology, and End Results Program single-year population estimates, stratified by sex and age in 10-y age groups (25–34, 35–44,…, 85+) [14]. Population projections to 2030 were sourced from the US Census Bureau 2012 National Population Projections [15]. Based on the number of annual CHD, stroke, and total CVD deaths (ICD codes I20–I51, I60–69) from 1979 to 2012 from the National Vital Statistics System [16], we projected mortality trends, by age and sex, to 2030 as previously described [17]. This allowed us to incorporate estimates of continuing declining trends in CVD mortality, rather than utilising only current CVD mortality rates as in other similar studies [18,19], a major advantage to avoid overestimating the benefits of any CVD intervention.

Data on age, sex, and SNAP participation status were acquired from the National Health Interview Survey (NHIS), 2000–2009. NHIS data were subsequently linked to mortality data from the Public-Use Linked Mortality Files (2000–2011), which provide follow-up for the NHIS sample through December 31, 2011. In total, we evaluated 499,740 adults aged ≥25 y who provided information on age, sex, and SNAP participation. Further details are published elsewhere [13]. This led to stratification by SNAP participation and eligibility: SNAP participants, SNAP-eligible non-participants, SNAP-ineligible individuals. Data on current consumption levels of F&Vs and SSBs, by age, sex, and SNAP status, were obtained from the nationally representative National Health and Nutrition Examination Survey (NHANES) 2009–2012 [20] using two consecutive 24-h dietary recalls, further incorporating projected intake forecasts derived from NHANES data from 1999–2012 [10] (S1 Appendix).

### Policy scenarios

The policy scenarios modelled were as follows: (a) a national MMC to increase F&V consumption and reduce SSB consumption targeting all American adults aged 25+ y, (b) a national tax to increase SSB prices by 10% (10% SSB tax), (c) a national subsidy to reduce F&V prices by 10% (10% F&V subsidy), (d) a SNAP-specific subsidy to reduce F&V prices by 30%, similar to the successful US Department of Agriculture (USDA) Healthy Incentives Pilot (HIP) in Massachusetts [21] (SNAP 30% F&V subsidy), and (e) a combination of all four policies above: a 10% SSB price increase for all, a 30% price reduction in total F&Vs for SNAP participants and a 10% price reduction for SNAP-eligible non-participants and SNAP-ineligible individuals, and a national F&V and SSB MMC for all (combined policy).

The baseline scenario of “no intervention” assumes that recent and current trends in consumption [10] simply continue. The combined policy scenario considers the associated change in dietary intake of each individual scenario as additive. However, the associated change in F&V intake for the 30% subsidy for SNAP participants is lower in the combined policy scenario than in isolation owing to different elasticities used for the universal (first 10% of the subsidy) and targeted (remaining 20% of the subsidy) aspects. This is explained further below.

### Association of price changes and dietary consumption (taxes and subsidies)

Extensive data from cross-sectional price elasticity and intervention studies support the association of changes in food or beverage prices with changes in consumption. We derived estimates from a recent meta-analysis of interventional and prospective longitudinal studies that directly evaluated the changes in consumption associated with changes in price [22]. In these studies, a 10% reduction in the price of F&Vs increased consumption by approximately 14% (95% CI 11%–17%), and a 10% increase in the price of SSBs reduced consumption by approximately 7% (95% CI 4%–10%). These pooled findings are broadly consistent with additional recent empirical evidence [12,23]. A recent evaluation of the USDA HIP showed that among SNAP-participating or -eligible populations, a 30% subsidy on the price of F&Vs purchased exclusively through the SNAP Electronic Benefits Transfer (EBT) card increased consumption by some 27% [21].

We recognised consistent evidence for differential associations by SES. For our national policies, we modelled a price elasticity gradient between SNAP participants and the SNAP-ineligible population of 50%, based on published estimates of the differential social associations of prices with food consumption [12,24]. We recognised that to achieve a 10% increase in national SSB prices, the actual implemented policy, e.g., an excise tax, might need to be modestly larger to offset any part of the tax that might be absorbed by industry. Thus, our findings should be interpreted as the likely association for the final retail price change of any tax or subsidy policy, and the specific policy formulation to achieve this price change could vary (e.g., agricultural subsidy, retailer subsidy, excise tax, sales tax). Methodology and sources are detailed further in S1 Appendix and S3 Table.

### Mass media campaign

We estimated the associated outcomes of a national MMC based on a meta-analysis of the association of national MMCs [25], including the US national 5 A Day campaign [26], with dietary habits, including F&V and SSB consumption. That pooled analysis indicated that a typical national MMC increases F&V intake and reduces SSB intake by approximately 7% each (95% CI 4%–9%). We further accounted for potentially varying coverage by age and sex using data from the 5 A Day campaign [26] (S1 Table). We modelled the associated outcomes of a 1-y MMC in 2015, assuming a 7% national association with increased F&V consumption at 1 y, which then fell linearly to a 20% residual association at year 5 (minimum estimate 5%, maximum estimate 40%), which then persisted to year 15 (i.e., 2030).

### Effects of dietary changes on cardiovascular disease

The effects of changes in consumption of F&Vs and SSBs upon CVD mortality were obtained from meta-analyses of prospective cohort studies and randomised trials [27] of the direct etiologic effects of fruit, vegetable, and SSB intake on CHD and stroke. These effect estimates are based on interventional and prospective observational studies (not exclusively randomised controlled trials [RCTs]). They are thus imperfect and should be interpreted as the best available evidence. The association sizes for the association of the policy components below (price changes and MMCs) with fruit, vegetable, and SSB consumption are aggregate estimates only, as provided in each respective meta-analysis, although we do include MMC coverage estimates stratified by age and sex taken from the evaluation of the 5 A Day campaign [26].

### The US IMPACT Food Policy Model

The US IMPACT Food Policy Model is an adaptation of the CHD IMPACT Model [3,28], and the IMPACT Food Policy Model has previously quantified potential health gains from healthier food policies in the UK [29–31] and Ireland [32]. The validated IMPACT methodology to calculate deaths prevented or postponed (DPPs) has been described [33] and is detailed in S1 Appendix. Briefly, using mortality trends (1979–2012), we estimated baseline mortality projections (no intervention) for each year from 2015–2030 for CHD and stroke [17] to provide the number of stroke and CHD deaths expected each year, stratified by age and sex. The crucial estimation quantifies the steadily declining CVD mortality rates in the US, and thus avoids substantial overestimation of the potential benefits of any preventive intervention [17]. To stratify these projections by SNAP status, we maintained the mortality rate ratio between each group throughout the 15-y projected period for baseline number of deaths.

We estimated the change in intake of F&Vs and SSBs by applying the appropriate intervention association measure to the baseline intake data for each stratum. We ran the model to calculate DPPs. We assumed equal coverage of price change policies among the relevant population in each scenario. The time lag from a price change policy being implemented to the subsequent change in F&V or SSB consumption was assumed to be less than a year; hence, no time lag was modelled. Finally, we assumed a sustained impact of the policy throughout the 15-y period, i.e., no attrition.

The US IMPACT Food Policy Model was used to calculate the expected change in numbers of CHD and stroke deaths attributable to changes in diet intake in our analysis. We first estimated the effect of each given policy scenario upon F&V and SSB intake. We then used the best evidence of effect size upon CHD and stroke for fruits, vegetables [27], and SSBs separately, stratified by age and sex. This provided the policy scenario association with CHD and stroke mortality, hence the “intervention expected number of deaths” following the intervention. The difference between the baseline and intervention expected deaths provided the cumulative DPPs from 2015 to 2030.

### Probabilistic sensitivity analyses

We used probabilistic sensitivity analysis to estimate the effect of uncertainty in key model parameters with given input probability distributions. For each policy scenario, we performed 10,000 iterations of the full model in R, version 3.2.2 [34], providing 95% UIs. The key parameters included the associated outcomes of the MMC, the association of price reduction with F&V intake and of price increase with SSB intake, consumption of F&Vs and SSBs, the effect size for the effect of F&V and SSB consumption upon CVD mortality individually, baseline CVD mortality, and ratio of ischaemic:haemorrhagic strokes. We varied each parameter by one standard deviation above and below the mean to assess the sensitivity of DPPs to each parameter in each scenario (S2 Fig). The F&V intake projection was the parameter generating the largest variation in DPPs when varied. Details on parameters and chosen distributions for the Monte Carlo simulation are available in S3 Table.

## Results

### Baseline demographics and disparities by SNAP status

There are approximately 21.6 million SNAP participant households (involving 44.5 million individual adults and children) nationwide and a further 20.9 million eligible but not participating in SNAP. The non-eligible population is approximately eight times larger, comprising approximately 173 million adults. Significant disparities in CVD mortality exist, with baseline aggregate age-standardised mortality more than 50% higher in SNAP participants compared to SNAP-ineligible adults (330/100,000 versus 206/100,000; using 2015 total population as reference) (Table 1), and even higher when only men are considered. There are also differences in the baseline intake of fruits, vegetables, and SSBs, with SNAP participants having consistently lower intake of F&Vs, and higher consumption of SSBs (Table 2). These differences are projected to persist to 2030.

**Table 1:** Population and cardiovascular disease mortality per 100,000 population by SNAP status, stratified by sex and age.

Sex and age group	All		SNAP participants		SNAP-eligible non-participants		SNAP-ineligible individuals	
	Population	Mortality	Population	Mortality	Population	Mortality	Population	Mortality
**All**								
Aggregate	215,861,896	221.1	21,635,836	330.0	20,902,917	256.4	173,323,143	205.7
25–34 y	43,940,831	3.7	6,274,629	4.7	4,708,692	4.7	32,957,510	3.3
35–44 y	40,386,647	17.2	4,646,631	36.7	3,642,194	18.9	32,097,823	14.2
45–54 y	43,017,876	57.1	3,926,325	162.1	3,421,058	67.5	35,670,493	44.5
55–64 y	40,821,192	135.9	3,467,339	284.7	3,791,909	229.9	33,561,944	110.0
65–74 y	27,494,977	310.0	1,893,703	606.5	2,381,429	335.6	23,219,845	283.2
75–84 y	13,894,175	877.5	967,571	973.3	2,000,427	924.6	10,926,176	860.4
85+ y	6,306,198	2,878.8	459,638	3,292.1	957,208	3,173.1	4,889,352	2,782.4
**Men**								
Aggregate	104,364,900	239.5	8,884,781	407.5	9,048,102	294.8	86,432,017	221
25–34 y	22,261,944	5.0	2,529,578	8.3	2,291,451	5.7	17,440,915	4.5
35–44 y	20,127,557	24.9	2,032,203	58.4	1,710,309	25.2	16,385,045	20.7
45–54 y	21,231,064	80.9	1,732,833	223.9	1,643,766	95.2	17,854,465	65.7
55–64 y	19,703,431	191.5	1,489,674	414.7	1,703,726	344.8	16,510,031	155.5
65–74 y	12,872,038	421.7	700,613	930.5	929,193	500.9	11,242,232	383.4
75–84 y	6,005,370	1,085.9	293,973	1,312.2	565,816	1,198.9	5,145,581	1,060.5
85+ y	2,163,496	3,211.4	105,907	3,878.3	203,841	3,545.5	1,853,748	3,136.5
**Women**								
Aggregate	111,496,996	203.9	12,751,055	290.4	11,854,815	239.2	86,891,126	187.2
25–34 y	21,678,887	2.3	3,745,051	2.3	2,417,241	3.8	15,516,595	2.0
35–44 y	20,259,090	9.6	2,614,428	19.8	1,931,885	13.3	15,712,778	7.5
45–54 y	21,786,812	33.9	2,193,492	113.3	1,777,292	41.9	17,816,028	23.4
55–64 y	21,117,761	84.1	1,977,665	186.8	2,088,183	136.2	17,051,913	65.8
65–74 y	14,622,939	211.7	1,193,090	416.2	1,452,236	229.9	11,977,613	189.1
75–84 y	7,888,805	718.9	673,598	825.3	1,434,611	816.4	5,780,595	682.3
85+ y	4,142,702	2,705.2	353,731	3,116.6	753,367	3,072.3	3,035,604	2,566.1

Aggregate values age-standardised using total US population as reference.

**Table 2:** Fruits, vegetable, and sugar-sweetened beverage consumption in SNAP participants, SNAP-eligible non-participants, and SNAP-ineligible individuals at baseline (2015) and for the baseline projection (2030) and all policies modelled, stratified by sex.

Scenario	Year	Consumption category	All					Men					Women				
			Total		SNAP participants	SNAP-eligible non-participants	SNAP-ineligible individuals	Total		SNAP participants	SNAP-eligible non-participants	SNAP-ineligible individuals	Total		SNAP participants	SNAP-eligible non-participants	SNAP-ineligible individuals
			Percent change	Consumption in grams/day (95% UI)				Percent change	Consumption in grams/day (95% UI)				Percent change	Consumption in grams/day (95% UI)			
**Baseline**	**2015**	**Fruits**		99.0 (79.0–122.0)	82.0 (50.0–124.0)	100.0 (73.0–131.0)	116.0 (77.0–163.0)		90.0 (64.0–122.0)	75.0 (35.0–135.0)	87.0 (53.0–130.0)	109.0 (58.0–176.0)		108.0 (80.0–141.0)	89.0 (45.0–149.0)	112.0 (73.0–160.0)	123.0 (69.0–192.0)
		**Vegs**		167.0 (141.0–196.0)	144.0 (100.0–198.0)	171.0 (136.0–211.0)	185.0 (134.0–246.0)		159.0 (123.0–200.0)	138.0 (79.0–215.0)	165.0 (117.0–222.0)	173.0 (106.0–258.0)		174.0 (138.0–218.0)	150.0 (90.0–228.0)	177.0 (127.0–235.0)	196.0 (126.0–287.0)
		**SSBs**		147.0 (121.0–175.0)	173.0 (122.0–232.0)	157.0 (121.0–196.0)	111.0 (71.0–160.0)		166.0 (128.0–209.0)	188.0 (114.0–281.0)	174.0 (123.0–237.0)	135.0 (74.0–214.0)		129.0 (96.0–166.0)	158.0 (93.0–241.0)	140.0 (96.0–193.0)	88.0 (42.0–152.0)
**Baseline projection**	**2030**	**Fruits**	13.0%	112.0 (91.0–137.0)	93.0 (58.0–137.0)	113.0 (85.0–146.0)	131.0 (89.0–181.0)	10.9%	100.0 (72.0–134.0)	83.0 (40.0–143.0)	97.0 (61.0–142.0)	120.0 (67.0–194.0)	14.7%	124.0 (93.0–159.0)	102.0 (53.0–168.0)	129.0 (89.0–179.0)	141.0 (83.0–214.0)
		**Vegs**	−0.4%	166.0 (140.0–195.0)	144.0 (100.0–195.0)	171.0 (135.0–211.0)	184.0 (134.0–245.0)	1.8%	161.0 (125.0–202.0)	140.0 (81.0–218.0)	168.0 (118.0–226.0)	176.0 (108.0–262.0)	−2.2%	171.0 (134.0–212.0)	147.0 (88.0–222.0)	173.0 (124.0–230.0)	192.0 (122.0–282.0)
		**SSBs**	−36.5%	93.0 (73.0–115.0)	110.0 (70.0–158.0)	100.0 (73.0–131.0)	70.0 (39.0–109.0)	−36.5%	105.0 (75.0–140.0)	119.0 (63.0–194.0)	111.0 (72.0–159.0)	86.0 (39.0–149.0)	−36.5%	82.0 (57.0–112.0)	101.0 (50.0–167.0)	89.0 (55.0–132.0)	56.0 (21.0–107.0)
**Media campaign**	**2030**	**Fruits**	14.1%	113.0 (92.0–137.0)	94.0 (59.0–138.0)	114.0 (86.0–147.0)	132.0 (91.0–184.0)	12.1%	101.0 (73.0–135.0)	84.0 (40.0–146.0)	98.0 (61.0–144.0)	122.0 (67.0–196.0)	15.7%	125.0 (94.0–161.0)	103.0 (54.0–169.0)	130.0 (88.0–179.0)	142.0 (84.0–218.0)
		**Vegs**	0.6%	168.0 (141.0–197.0)	145.0 (100.0–197.0)	172.0 (136.0–214.0)	186.0 (135.0–247.0)	2.9%	163.0 (126.0–205.0)	142.0 (82.0–219.0)	170.0 (121.0–229.0)	178.0 (110.0–265.0)	−1.3%	172.0 (135.0–215.0)	148.0 (87.0–225.0)	175.0 (126.0–233.0)	194.0 (123.0–283.0)
		**SSBs**	−37.2%	92.0 (73.0–116.0)	108.0 (69.0–156.0)	98.0 (72.0–130.0)	70.0 (40.0–110.0)	−37.2%	104.0 (75.0–139.0)	118.0 (63.0–193.0)	109.0 (70.0–159.0)	85.0 (39.0–151.0)	−37.1%	81.0 (57.0–111.0)	100.0 (50.0–165.0)	88.0 (54.0–131.0)	55.0 (22.0–109.0)
**10% SSB tax**	**2030**	**Fruits**	13.0%	112.0 (91.0–136.0)	93.0 (58.0–136.0)	113.0 (85.0–146.0)	131.0 (90.0–183.0)	10.9%	100.0 (72.0–133.0)	83.0 (39.0–144.0)	97.0 (61.0–140.0)	120.0 (66.0–190.0)	14.7%	124.0 (93.0–159.0)	102.0 (53.0–166.0)	129.0 (88.0–177.0)	141.0 (82.0–217.0)
		**Vegs**	−0.4%	166.0 (140.0–195.0)	144.0 (100.0–194.0)	171.0 (135.0–211.0)	184.0 (133.0–243.0)	1.8%	161.0 (125.0–202.0)	140.0 (81.0–217.0)	168.0 (119.0–226.0)	176.0 (107.0–259.0)	−2.2%	171.0 (134.0–212.0)	147.0 (87.0–221.0)	173.0 (125.0–230.0)	192.0 (123.0–281.0)
		**SSBs**	−41.3%	86.0 (68.0–107.0)	101.0 (65.0–146.0)	92.0 (66.0–121.0)	66.0 (37.0–103.0)	−41.3%	97.0 (69.0–130.0)	110.0 (57.0–179.0)	102.0 (65.0–148.0)	80.0 (38.0–138.0)	−41.3%	76.0 (53.0–104.0)	93.0 (47.0–154.0)	82.0 (51.0–122.0)	52.0 (20.0–101.0)
**10% F&V subsidy**	**2030**	**Fruits**	29.7%	129.0 (104.0–156.0)	107.0 (67.0–156.0)	131.0 (98.0–168.0)	149.0 (102.0–207.0)	27.3%	115.0 (82.0–153.0)	96.0 (46.0–167.0)	111.0 (69.0–162.0)	137.0 (74.0–218.0)	31.6%	142.0 (108.0–184.0)	117.0 (62.0–194.0)	149.0 (101.0–206.0)	160.0 (95.0–247.0)
		**Vegs**	14.4%	191.0 (161.0–224.0)	165.0 (115.0–227.0)	197.0 (155.0–241.0)	210.0 (153.0–279.0)	16.8%	185.0 (143.0–233.0)	162.0 (93.0–252.0)	194.0 (137.0–260.0)	200.0 (123.0–299.0)	12.3%	196.0 (153.0–244.0)	169.0 (101.0–258.0)	199.0 (142.0–265.0)	219.0 (139.0–320.0)
		**SSBs**	−36.5%	93.0 (73.0–116.0)	110.0 (70.0–158.0)	100.0 (72.0–132.0)	70.0 (40.0–109.0)	−36.5%	105.0 (76.0–140.0)	119.0 (63.0–192.0)	111.0 (70.0–161.0)	86.0 (40.0–150.0)	−36.5%	82.0 (56.0–113.0)	101.0 (50.0–168.0)	89.0 (54.0–132.0)	56.0 (22.0–109.0)
**SNAP 30% F&V subsidy**	**2030**	**Fruits**	23.2%	122.0 (98.0–149.0)	123.0 (76.0–180.0)	113.0 (85.0–146.0)	131.0 (88.0–181.0)	21.0%	109.0 (78.0–147.0)	110.0 (52.0–192.0)	97.0 (60.0–140.0)	120.0 (66.0–194.0)	24.9%	135.0 (101.0–175.0)	135.0 (70.0–222.0)	129.0 (87.0–178.0)	141.0 (82.0–216.0)
		**Vegs**	9.0%	182.0 (152.0–215.0)	190.0 (130.0–261.0)	171.0 (135.0–211.0)	184.0 (132.0–244.0)	11.4%	177.0 (136.0–223.0)	186.0 (107.0–289.0)	168.0 (119.0–225.0)	176.0 (107.0–261.0)	6.9%	186.0 (145.0–235.0)	194.0 (113.0–298.0)	173.0 (124.0–230.0)	192.0 (121.0–283.0)
		**SSBs**	−36.5%	93.0 (73.0–116.0)	110.0 (70.0–158.0)	100.0 (71.0–132.0)	70.0 (40.0–111.0)	−36.5%	105.0 (75.0–141.0)	119.0 (63.0–194.0)	111.0 (70.0–161.0)	86.0 (40.0–153.0)	−36.5%	82.0 (57.0–112.0)	101.0 (51.0–167.0)	89.0 (54.0–132.0)	56.0 (22.0–109.0)
**Combined**	**2030**	**Fruits**	41.7%	141.0 (115.0–170.0)	123.0 (77.0–180.0)	150.0 (111.0–195.0)	149.0 (102.0–206.0)	38.8%	125.0 (90.0–166.0)	110.0 (53.0–190.0)	128.0 (80.0–190.0)	137.0 (74.0–217.0)	43.9%	155.0 (118.0–200.0)	135.0 (71.0–222.0)	171.0 (116.0–239.0)	160.0 (93.0–244.0)
		**Vegs**	25.2%	209.0 (176.0–245.0)	190.0 (131.0–261.0)	226.0 (180.0–279.0)	210.0 (152.0–279.0)	28.0%	203.0 (158.0–256.0)	186.0 (107.0–295.0)	223.0 (157.0–301.0)	200.0 (121.0–297.0)	22.8%	214.0 (168.0–267.0)	194.0 (115.0–293.0)	229.0 (165.0–308.0)	219.0 (139.0–320.0)
		**SSBs**	−41.9%	85.0 (67.0–106.0)	100.0 (63.0–145.0)	91.0 (66.0–120.0)	65.0 (37.0–102.0)	−42.0%	96.0 (69.0–128.0)	109.0 (57.0–177.0)	101.0 (64.0–146.0)	79.0 (36.0–140.0)	−41.9%	75.0 (52.0–103.0)	92.0 (46.0–153.0)	81.0 (50.0–121.0)	52.0 (19.0–100.0)

Actual and forecasted consumption by SNAP group in grams/day. Percent change is relative change of estimated consumption for given scenario versus 2015 baseline consumption values.

F&V, fruit and vegetable; SNAP, Supplemental Nutrition Assistance Program; SSB, sugar-sweetened beverage; UI, uncertainty interval; Vegs, vegetables.

### Associations of policy scenarios with dietary intake

The change potentially achievable in consumption of F&Vs varies substantially across the different policy scenarios, and across the SNAP groups. By 2030, the national 10% F&V subsidy would increase aggregate fruit consumption by 15% compared to baseline (129 g/d versus 112 g/d), some 17 times more than the national MMC (Table 2), with a similar association for vegetable consumption. The national 10% SSB tax would reduce SSB consumption by 8% on average, whilst the national 10% F&V subsidy would increase F&V consumption considerably in all groups. Consumption of fruits among SNAP participants in 2030 is projected to remain 28% lower than among SNAP-ineligible individuals (107 g/d versus 149 g/d). This difference is projected to be similar for consumption of vegetables. With the 30% F&V targeted subsidy for SNAP participants, the projected difference in fruit consumption is reduced to just 6% (Table 2).

### Cardiovascular disease mortality

**Fig 1:**
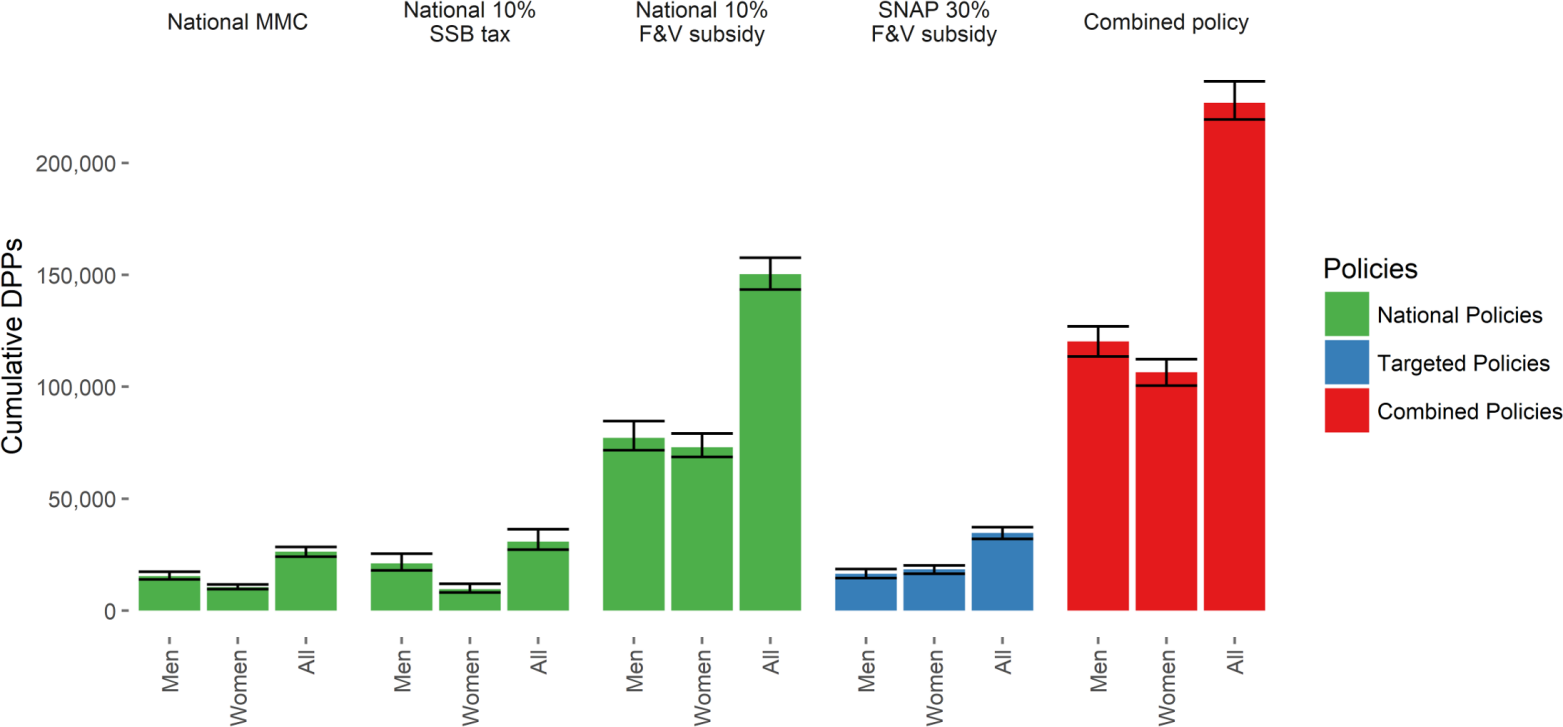
Cumulative deaths prevented or postponed from 2015 to 2030 under each policy modelled, by sex. Error bars indicate 95% uncertainty intervals. DPPs, deaths prevented or postponed; F&V, fruit and vegetable; MMC, mass media campaign; SNAP, Supplemental Nutrition Assistance Program; SSB, sugar-sweetened beverage.

The 10% F&V subsidy is the most effective single policy in reducing CVD mortality over the period 2015–2030. This policy could yield approximately 150,500 DPPs (95% UI 141,400–158,500). This mortality reduction comprises some 78,100 DPPs from CHD, and 72,400 from stroke, reducing the overall mortality rate by approximately 4/100,000 against baseline (Table 3; Fig 1). These mortality reductions are some seven times higher than those projected for the other national policies. The 10% SSB tax could reduce CVD mortality by approximately 31,000 DPPs (95% UI 26,800–35,300). This would reduce the CVD mortality rate by 0.8/100,000. Finally, the MMC, targeting both F&V and SSB consumption, would have slightly less effectiveness, representing some 25,800 (95% UI 24,300–28,500) DPPs coming equally from CHD and stroke DPPs. The MMC policy could reduce CVD mortality by approximately 0.7/100,000. However, the majority of DPPs would be gained in the first year of the media policy, after which the benefit would fall substantially. The 30% F&V targeted subsidy for SNAP participants could reduce CVD mortality by approximately 35,100 DPPs (95% UI 31,700–37,500), representing a 0.9/100,000 decrease in mortality rate. The combined policy would be more effective than any single policy, generating approximately 230,000 DPPs (95% UI 215,800–237,100) over the 15-y period. This would comprise approximately 90,700 stroke DPPs and 137,300 CHD DPPs, reducing the CVD mortality rate by approximately 6.1/100,000 (Table 3).

**Table 3:** Total cumulative cardiovascular disease deaths prevented or postponed from 2015 to 2030 under each policy modelled, stratified by cardiovascular disease subtype and sex.

Scenario	Measure	Coronary heart disease aggregate	Stroke aggregate	Cardiovascular disease		
				Aggregate	Men	Women
**Media campaign**	DPPs	17,000 (15,600–19,200)	8,800 (8,000–10,100)	25,800 (24,300–28,500)	15,400 (14,000–17,500)	10,400 (9,600–11,900)
	DPPs/100,000	0.46 (0.42–0.52)	0.24 (0.22–0.27)	0.69 (0.65–0.76)	0.85 (0.78–0.97)	0.54 (0.50–0.62)
**10% SSB tax**	DPPs	31,000 (26,800–35,300)	0 (0–0)	31,000 (26,800–35,300)	21,200 (17,600–25,100)	9,800 (7,900–11,900)
	DPPs/100,000	0.83 (0.72–0.95)	0.00 (0.00–0.00)	0.83 (0.72–0.95)	1.18 (0.97–1.39)	0.51 (0.41–0.62)
**10% F&V subsidy**	DPPs	78,100 (72,000–84,300)	72,400 (66,200–77,800)	150,500 (141,400–158,500)	77,300 (70,900–83,500)	73,100 (67,200–78,600)
	DPPs/100,000	2.10 (1.94–2.27)	1.95 (1.78–2.09)	4.04 (3.80–4.26)	4.28 (3.92–4.63)	3.82 (3.51–4.11)
**SNAP 30% F&V subsidy**	DPPs	20,000 (17,600–22,200)	15,100 (13,100–16,500)	35,100 (31,700–37,500)	16,700 (14,400–18,500)	18,400 (16,200–20,200)
	DPPs/100,000	0.54 (0.47–0.60)	0.41 (0.35–0.44)	0.94 (0.85–1.01)	0.92 (0.80–1.03)	0.96 (0.85–1.06)
**Combined**	DPPs	137,300 (128,100–145,400)	90,700 (83,600–96,000)	228,000 (215,800–237,100)	120,500 (111,700–128,200)	107,600 (100,100–113,600)
	DPPs/100,000	3.69 (3.44–3.91)	2.44 (2.25–2.58)	6.13 (5.80–6.37)	6.67 (6.18–7.10)	5.62 (5.23–5.93)

95% uncertainty intervals given in parentheses. DPPs rounded to nearest 100. DPPs/100,000 rounded to two decimal places.

DPPs, deaths prevented or postponed; F&V, fruit and vegetable; SNAP, Supplemental Nutrition Assistance Program; SSB, sugar-sweetened beverage.

### Cardiovascular disease socio-economic disparities

**Fig 2:**
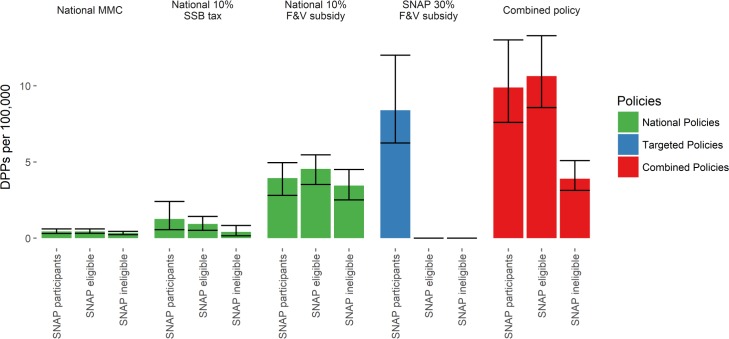
Deaths prevented or postponed per 100,000 population for each policy modelled in 1 y—2030—by SNAP group. Error bars indicate 95% uncertainty intervals. DPPs, deaths prevented or postponed; F&V, fruit and vegetable; MMC, mass media campaign; SNAP, Supplemental Nutrition Assistance Program; SSB, sugar-sweetened beverage.

In absolute terms, of the individual policies, the targeted SNAP 30% F&V subsidy would yield the largest number of averted deaths in SNAP participants, yielding approximately 35,100 DPPs (95% UI 31,700–37,500) (Table 4; Fig 2). This represents a reduction in CVD mortality of 9.5/100,000 in this group compared to those ineligible for SNAP. This is more than two times, five times, and 11 times more DPPs that would be generated in the SNAP group compared with the universal 10% F&V subsidy, 10% SSB tax, and media campaign, respectively. In relative terms, the targeted SNAP 30% F&V subsidy would also be the most effective in reducing CVD disparities. All of the national policies would reduce CVD disparities between SNAP participants and the SNAP-ineligible population, but less than the targeted 30% F&V subsidy. The national 10% F&V subsidy and 10% SSB tax were approximately three and five times more effective at reducing disparities than the media campaign (Table 4). The combined policy is effective at reducing disparities: this scenario generates 11.6 DPPs per 100,000 in SNAP participants, some 7 DPPs per 100,000 more than in the SNAP-ineligible population.

**Table 4:** Total cumulative deaths prevented or postponed from 2015 to 2030 under each policy modelled, stratified by SNAP group.

Scenario	Measure	SNAP participants	SNAP-eligible non-participants	SNAP-ineligible individuals	Aggregate
**Media campaign**	DPPs	3,200 (3,000–3,700)	3,300 (3,100–3,700)	19,300 (17,700–21,700)	25,800 (24,300–28,500)
	DPPs/100,000	0.88 (0.80–0.99)	0.92 (0.86–1.01)	0.64 (0.59–0.73)	0.69 (0.65–0.76)
**10% SSB tax**	DPPs	6,700 (5,600–7,700)	5,300 (4,600–5,900)	19,100 (15,200–23,300)	31,000 (26,800–35,300)
	DPPs/100,000	1.81 (1.52–2.10)	1.45 (1.26–1.64)	0.64 (0.51–0.78)	0.83 (0.72–0.95)
**10% F&V subsidy**	DPPs	16,700 (15,300–18,000)	19,700 (18,400–20,700)	114,100 (105,500–122,200)	150,500 (141,400–158,500)
	DPPs/100,000	4.54 (4.17–4.87)	5.41 (5.06–5.70)	3.82 (3.53–4.09)	4.04 (3.80–4.26)
**SNAP 30% F&V subsidy**	DPPs	35,100 (31,700–37,500)	0	0	35,100 (31,700–37,500)
	DPPs/100,000	9.53 (8.62–10.17)	0	0	0.94 (0.85–1.01)
**Combined**	DPPs	42,900 (39,300–45,600)	47,500 (44,300–49,700)	137,600 (127,400–146,800)	228,000 (215,800–237,100)
	DPPs/100,000	11.64 (10.67–12.37)	13.07 (12.19–13.67)	4.61 (4.26–4.91)	6.13 (5.80–6.37)

95% uncertainty intervals given in parentheses. DPPs rounded to nearest 100. DPPs/100,000 rounded to nearest two decimal places.

DPPs, deaths prevented or postponed; F&V, fruit and vegetable; SNAP, Supplemental Nutrition Assistance Program; SSB, sugar-sweetened beverage.

**Fig 3:**
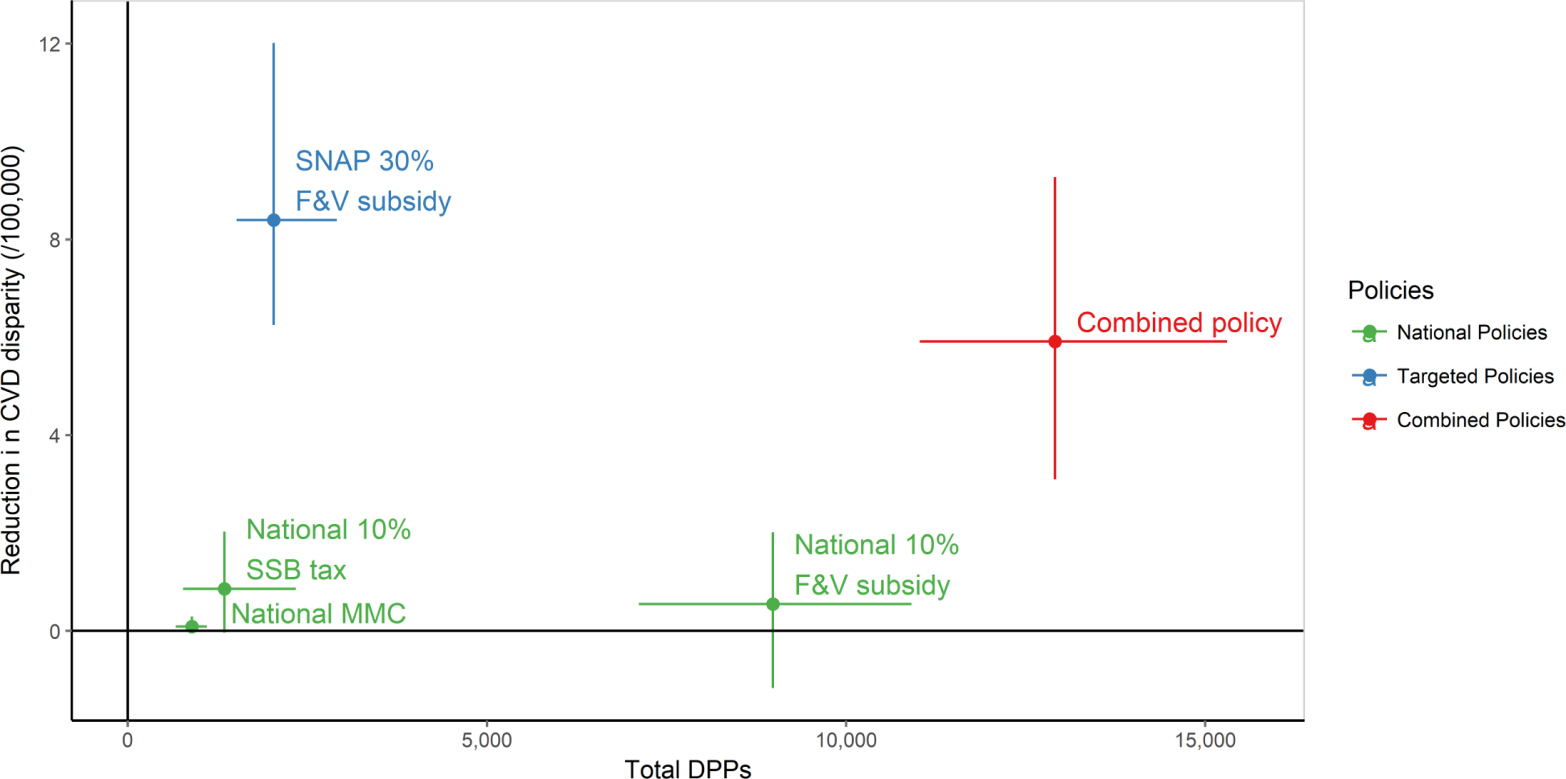
Total deaths prevented or postponed versus change in cardiovascular disease disparities in 1 y—2030. The reduction in CVD disparities is the difference in DPPs/100,000 population between SNAP participants and SNAP-ineligible individuals (a positive number indicates more DPPs/100,000 in SNAP participants than in SNAP-ineligible individuals). Point estimate and 95% uncertainty intervals. CVD, cardiovascular disease; DPPs, deaths prevented or postponed; F&V, fruit and vegetable; MMC, mass media campaign; SNAP, Supplemental Nutrition Assistance Program; SSB, sugar-sweetened beverage.

**Fig 4:**
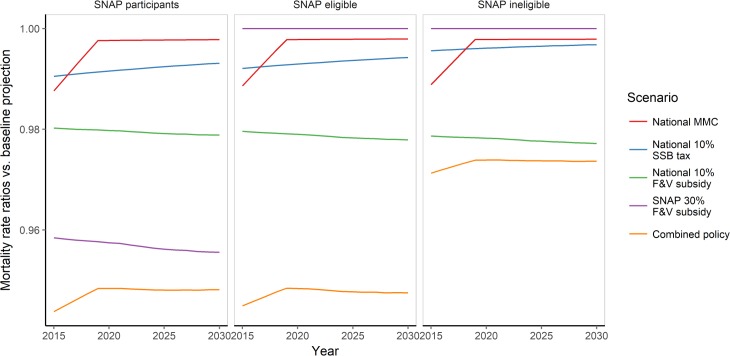
Standardised cardiovascular disease mortality rate ratio of each policy scenario versus baseline projection (reference) from 2015 to 2030 by SNAP group. Mortality rate ratio demonstrates mortality rate in 2030 under each given policy scenario versus baseline projection mortality rate. F&V, fruit and vegetable; MMC, mass media campaign; SNAP, Supplemental Nutrition Assistance Program; SSB, sugar-sweetened beverage.

**Fig 5:**
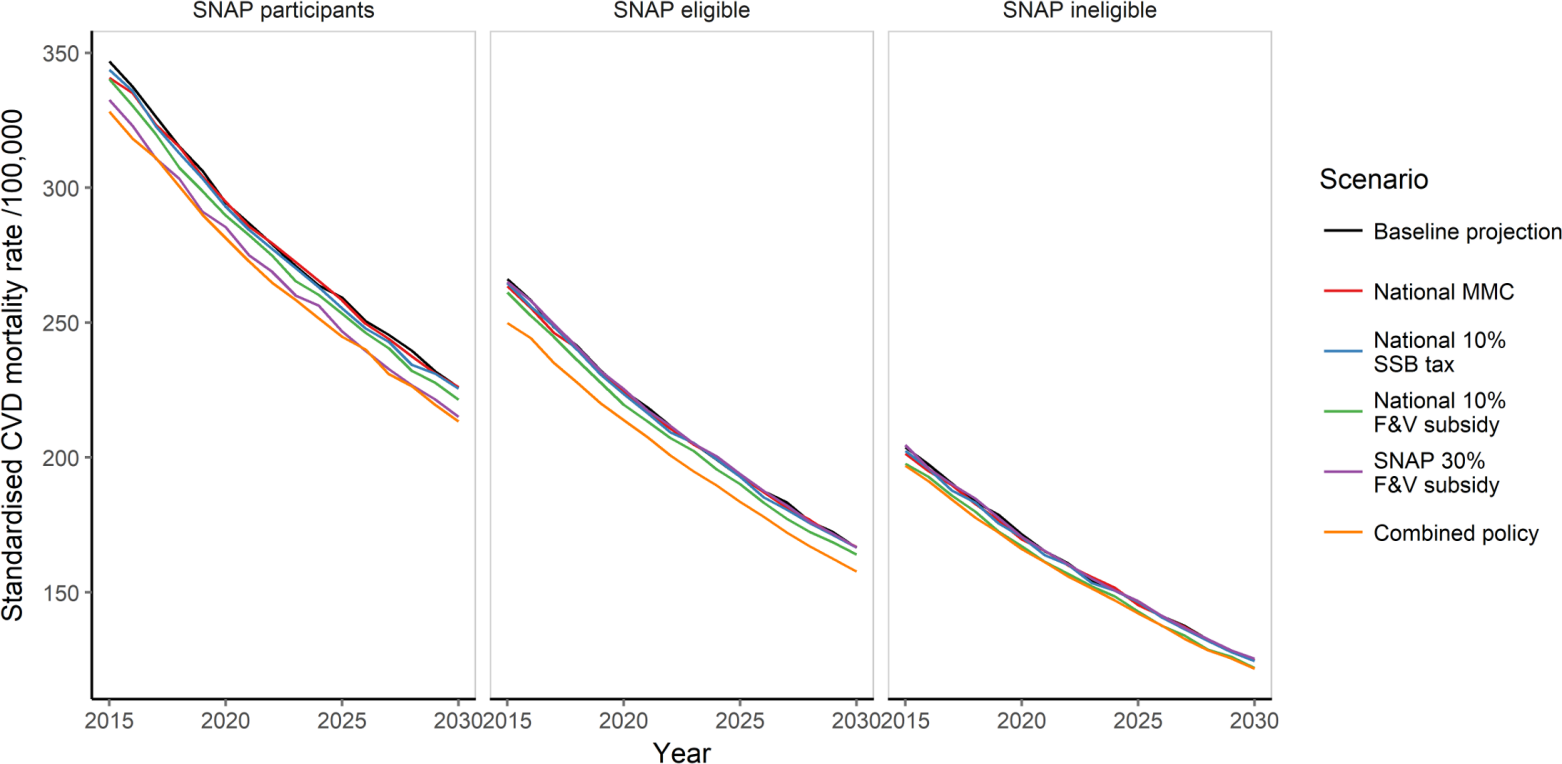
Standardised cardiovascular disease mortality rate (per 100,000 population) from 2015 to 2030 under for baseline projection and all policy scenarios, by SNAP group. CVD, cardiovascular disease; F&V, fruit and vegetable; MMC, mass media campaign; SNAP, Supplemental Nutrition Assistance Program; SSB, sugar-sweetened beverage.

The combined policy might best achieve the goal of reducing both the overall mortality burden and CVD disparities (Table 4; Fig 3). This approach could potentially generate approximately 12,800 DPPs in 2030 and thus reduce CVD disparities by approximately 6.0 deaths per 100,000. Whilst the national 10% F&V subsidy might generate almost 8,900 DPPs in the year 2030 alone, its effectiveness in reducing CVD disparities would be substantially lower than that of the combined policy. In contrast, although the targeted 30% F&V subsidy could generate substantially fewer DPPs than the national 10% F&V subsidy (approximately 2,100) in 2030, this might be the most effective policy for reducing CVD disparities (by approximately 8.5 deaths per 100,000 in 1 y) (Table 4; Figs 3–5).

## Discussion

Our study suggests that reducing the price of healthy foods for SNAP participants whilst additionally reducing SSB consumption nationally through taxes and a media campaign could potentially reduce socio-economic disparities in CVD mortality and powerfully improve dietary quality, the leading risk factor for CVD.

This is the first US study to our knowledge to compare the likely effects of national policies targeting F&V and SSB consumption and a policy targeting F&V consumption in the SNAP population upon CVD mortality and socio-economic disparities. Policies effectively increasing F&V consumption or reducing SSB consumption might powerfully reduce CVD mortality and disparities. Further, a combination of these policies could be even more powerful. Whilst all four individual policies would result in reductions in deaths by 2030, the magnitude and rate of such reductions differed substantially. The 10% F&V subsidy might reduce CVD mortality by some 2.1%, saving approximately 150,500 deaths from 2015 to 2030. It might thus be approximately five times more effective than the MMC. The SNAP 30% F&V subsidy might be four times less effective at reducing total US CVD mortality than the national 10% F&V subsidy, but could be the most effective approach in reducing CVD socio-economic disparities.

The findings of this study have important implications for crafting specific price and incentive policy approaches to optimise access to SSBs and F&Vs, respectively. F&Vs have high production costs because certain crops are especially susceptible to adverse weather, have limited storage time, often have to be transported with temperature control, or typically have to be hand-picked or hand-sorted [35]. The ultimate price that consumers pay for F&Vs is affected by policies and practices that have impact across the entire food production system [36], including international trade agreements, immigration law, import/export policies, as well as the technology used to harvest and transport fragile crops across the globe. Embedding pricing incentives systematically within government feeding programmes such as SNAP could increase the purchase and consumption of F&Vs within low-income populations. These benefits could be extended if Electronic Benefits Transfer was integrated into all farmers markets, allowing recipients to authorise transfer of their government benefits to the retailers in local markets. Retail outlets where consumers make their final purchase are playing an increasingly important role in food pricing. Non-traditional retailers/discount stores are making F&Vs more affordable. There are also efforts underway by large retailers to encourage local sustainable agriculture to support the availability and affordability of fresh fruits, vegetables, and other specialty crops in their stores. Other pricing-related policy approaches could extend to growers, providing them with more accessible crop insurance, agricultural subsidies for growing specialty crops, or government incentives to diversify crops across base acres of land. Such programmes could be financed through revenue raised by the modelled 10% SSB price increase or other public health taxes. Thus, a system-wide approach to price strategies might be particularly effective for improving diet [37].

The differing effectiveness in reducing aggregate CVD mortality across the four individual policies can be attributed to several factors. F&V consumption at baseline was higher than SSB consumption, and the elasticity of a F&V subsidy is greater than that of a SSB price increase; with these factors coupled together, a 10% price reduction for F&Vs results in a greater change in consumption than a 10% price increase for SSBs. Further, SSB consumption is highest in younger age groups, where CVD mortality is low. Whilst the 10% SSB price increase has an equally large proportional association with consumption across age groups, the differences in baseline consumption across age groups result in smaller absolute dietary reductions in the middle and older age groups, which experience the majority of the CVD burden. The apparent effectiveness of each policy also varied by SNAP group. Baseline CVD mortality is approximately 60% higher in SNAP participants [13] compared to those not eligible for SNAP. SNAP participants are also more price sensitive. Despite this, the F&V subsidy resulted in a larger absolute increase in consumption of F&Vs in SNAP-eligible non-participants and SNAP-ineligible individuals. This was due to the consistently large relative association with consumption (14% increase) and baseline F&V intake being much higher in these two groups.

### Public health policy implications

Low intake of F&Vs is a risk factor for CVD as well as certain cancers, and intake is often lowest in the most deprived groups in society, thus widening disparities. In the US between 1999 and 2012, F&V consumption remained [10] substantially below the recommended amounts of 2.5 cups of vegetables and 2 cups of fruit per day [38], while disparities by income, education, and race/ethnicity did not improve. SSB intake declined by only about half a serving per day during this period, thus remaining high and with significant remaining disparities [10].

Public health strategies can aim to improve the environment (“structural policies”) or facilitate behaviour change in individuals using their personal resources (“agentic policies”) or do both [39]. Most national governments currently favour agentic policies for dietary change, rather than population-level structural policies [40]. This can be contrasted to many other existing government health- and safety-focused policies and standards that strongly favour structural approaches, such as biosafety and food contaminants, water and air safety, and toy, motor vehicle, housing, and occupational safety. Structural interventions, which are not dependent on individual responses, are generally the most effective and sustainable, as well as being the most equitable [39,40]. One additional important policy, not modelled here, would be to consider removing SSBs from SNAP-eligible items. Our findings suggested that a combined national fiscal intervention, enhanced by additional intervention among those with lower resources and worse diet and health, would be most effective in reducing the dual burdens of CVD mortality and socio-economic disparities. These results are consistent with the notion of “proportionate universalism” [41,42], and lend support to the idea that a combination of structural and agentic policies appears more effective for reducing the unequal burden of CVD in populations [40]. We did not model policy costs, nor the cost-effectiveness of the policy scenarios. However, not accounting for potential savings, the subsidies are likely to cost more than the media campaign to implement and run, whilst the SSB tax would be revenue-raising; hence, combining this tax with a subsidy might make the policy fiscally neutral. These novel results might be useful to inform policy makers in the US, such as those developing the new USDA Farm Bill, which includes SNAP, as well as leaders in professional advocacy associations such as the American Heart Association.

### Strengths and limitations

This study has several strengths. We used nationally representative datasets encompassing the US adult population [14,16]. Further, we used comprehensive meta-analyses for effect sizes for the effect of F&V [27] and SSB consumption upon CVD mortality and for the associations of each policy with F&V intake within the US population [22,25]. The effect size for the effect of SSB consumption upon CVD accounts for direct effects upon CHD only and hence may underestimate the total effects, which include potential effects upon stroke mediated by change in body mass index. Using the HIP elasticity [21] for a targeted F&V price reduction was helpful, as was stratifying potential policy associations by SNAP group. Further, our health outcomes analysis sensibly assumes that the recent declines in CVD mortality will likely continue [17], unlike more conventional methods, which simply use a static baseline. If, in future, mortality rates plateau (as already seen in young adults [43]) or even increase [43,44], mortality savings from the modelled policies would be even greater. Despite our baseline mortality projections accounting for recent trends in CVD mortality, we do not account for competing risks of other diseases such as cancers over the 15-y period modelled, nor additional change in the policy environment over this period.

This study also has limitations. The effect of F&V and SSB consumption upon CHD and stroke mortality, and the association of each policy with consumption, are taken from comprehensive meta-analyses of interventional and prospective observational studies; they are thus imperfect estimates [22,25,27,45] compared to dietary RCT-level evidence such as that from the PREDIMED trial [46]. Further, an increase in consumption of F&Vs would have further benefits upon incidence of diabetes and some cancers not modelled here. We also assumed a short lag time between policy implementation and reductions in CVD mortality. However, evidence consistently supports this assumption [47]. We did not account for additional (new) dietary policies being implemented over the 15-y period such as those targeting salt consumption, an important CVD risk factor. Dietary policies targeting salt have been effectively implemented in several countries and the US Food and Drug Administration has proposed a voluntary reformulation strategy. Modelling the potential health effects of the proposed reformulation would be of real use to policy makers. Our model analyses potential effects of food policies upon CVD mortality and disparities in CVD mortality; however, such policies would undoubtedly have effects upon CVD incidence and CVD health care costs as well. Whilst we incorporated coverage estimations by age and sex for the MMC using data from the nationwide 5 A Day campaign [26], less information exists regarding the decaying impact of media campaigns. We therefore assumed an approximate 20% residual effect after 5 y. However, this assumption was tested robustly, using wide uncertainty parameters (5%–40%). We estimated a gradient of price elasticity for SSBs and F&Vs between SNAP participants and the population not participating in SNAP using the best available evidence [12,21]. However, we lacked the detailed data needed to stratify for differing price elasticities of demand by age or sex. This could therefore lead to underestimation of the association of changes in consumption from such policies and reduction in socio-economic disparities. Similarly, we did not report life years gained and hence may have under-reported the associated outcomes of these policies for disparities, given the younger average age of CVD incidence in lower income groups [13]. We did not explicitly account for any substitution effects when increasing F&V consumption; however, the meta-analyses from which model parameters were derived used observed effects, thus accounting for average actual population substitutes and compliments. Furthermore, focused efforts to encourage specific substitutions could make such interventions even more effective. Future research addressing the cost-effectiveness of such specific food policies is also warranted.

### Conclusions

Fiscal strategies targeting diet might substantially help to reduce the unequal CVD mortality burden in the US. All four individual dietary policies could be effective, whilst a combination of national and targeted policies might be even more powerful in reducing both CVD mortality and socio-economic disparities.

## Supporting information

S1 Appendix US IMPACT Food Policy Model—modelling health outcomes. (DOC)

S1 Fig Schematic outlining the US IMPACT Food Policy Model. (TIFF)

S2 Fig Tornado plot showing the contribution of different model parameters to overall uncertainty. (TIFF)

S1 Table Coverage estimates of the mass media campaign stratified by age and sex. (DOC)

S2 Table Expected deaths (baseline deaths) and cumulative deaths prevented or postponed and cardiovascular disease mortality reduction from 2015 to 2030 achieved through all policies modelled. (DOC)

S3 Table Probabilistic sensitivity analysis in the US IMPACT Food Policy Model parameters. (DOC)

S4 Table Key assumptions for the US IMPACT Food Policy Model. (DOC)

## Abbreviations

CHD: coronary heart disease
CVD: cardiovascular disease
DPPs: deaths prevented or postponed
F&V: fruit and vegetable
HIP: Healthy Incentives Pilot
MMC: mass media campaign
NHANES: National Health and Nutrition Examination Survey
NHIS: National Health Interview Survey
RCT: randomised controlled trial
SES: socio-economic status
SNAP: Supplemental Nutrition Assistance Program
SSB: sugar-sweetened beverage
UI: uncertainty interval
USDA: US Department of Agriculture
