# Supplementary material for: Reducing US cardiovascular disease burden and disparities through national and targeted dietary policies: A modelling study
Source: PLoS Med. 2017 Jun 6;14(6):e1002311. doi: 10.1371/journal.pmed.1002311 (PMC5460790; doi:10.1371/journal.pmed.1002311)
Supplement: S1 Appendix — (DOC) [file pmed.1002311.s001.doc]

**S1 Appendix**

**US IMPACT Food Policy Model –** *modeling health outcomes*

**Fruits and vegetable projected trends**

Briefly, for each stratum we fit a survey-weighted linear regression model with survey year as the independent variable and fruit/vegetable intake as the dependent variable. This provided intake estimates for each stratum for 2015-2030. The standard error of the forecasted intakes accounted for prediction out of sample (e.g. intake estimates became less precise the further out of sample we predicted). There was no evidence of non-linear trends in any strata. Sensitivity analyses were conducted.

**Estimating the effect of change in price upon consumption of fruits and vegetables**

In the regression approach methodology used, the regression coefficient for reduction in price of F&V (14% increase consumption per 10% price reduction)[1] is applied to baseline fruit and vegetable intake in a given year (eg 2016) for each age, gender and race strata. This produces resulting F&V consumption post intervention.

**Different price elasticity by socio-economic status group**

Green et al[2] provides elasticity estimates for food groups by income strata – ‘low’, ‘medium’ and ‘high’. The elasticity estimate for ‘all foods’ varies from -0.91 in the low income group, to -0.77 in the high income group. This suggests that the elasticity for the low-income group is 1.182 (18.2%) higher than in the high-income group. We therefore applied an 18.2% higher elasticity to the SNAP groups (lowest income) compared to the SNAP ineligible group (high-income) in our analysis.

**Estimating the effect of mass media campaign upon consumption of fruits and vegetables and SSBs**

We used the regression coefficient for mass media campaign (7% increase consumption[3, 4]) and multiplied this by the coverage estimate of the MMC. The coverage estimate is a number between 0 and 1 to reflect differing efficacy of the MMC by age, gender and race. The coverage estimates were derived from the US ‘5 a day’ evaluation[5]. This process gave a strata (age and gender) specific regression coefficient for effectiveness of the MMC upon change in F&V consumption.

**Estimating the effect of change in price upon consumption of SSBs**

In the regression approach methodology used, the regression coefficient for increase in price of SSB (7% reduced consumption per 10% price reduction)[1] is applied to baseline SSB intake in a given year (eg 2016) for each age, gender and SNAP group strata. This produces resulting SSB consumption post intervention.

**Estimating the effect of change in F&V consumption upon CVD mortality**

The change in F&V consumption is calculated by subtracting consumption post intervention from baseline consumption for each year and each age, gender and SNAP strata. We then calculate a subgroup (age, gender and SNAP) specific regression coefficient for the given change in Fruits and vegetable consumption separately using regression coefficients from Micha et al[6].

We then apply the strata specific (age and gender) regression coefficient calculated in the previous step to the baseline number of deaths in that specific strata for each given year in the study period (2015-2030). This provides the difference between the baseline number of deaths and the intervention expected number of deaths. This difference we call ‘Deaths Prevented or Postponed’ (DPPs). The DPPs from each strata over the study period are then summed to give total cumulative DPPs over the study period.

**Example: Estimating the numbers of death prevented or postponed by changes in fruits and vegetables consumption through a national 10% price reduction:**

***CVD Mortality fall due to increased consumption of fruits and vegetables in men aged 65-74***

For example, from 2015 to 2030, 239,600 CVD deaths would be expected among men aged 45-54 years in the US. The effect size of the price change was taken from Afshin et al[1], with a 14% increase consumption of fruits and vegetables for 10% price decrease. The subsequent change in consumption was applied to regression coefficient for effect of F&V (separately) intake upon CHD and stroke mortality from Micha et al[6] treating it as loglinear. This leads to aggregate CHD and stroke mortality regression coefficients for the policy scenario. This is then applied to the expected number of deaths in each year in the study period (2015-2030) giving a reduction in deaths, or Deaths Prevented or Postponed (DPP) figure for each year. These are then summed to give cumulative DPPs over the study period.

We treated the meta-analysis relationship between fruits and vegetables (separately) consumption and CHD and stroke (incidence) mortality[6] as log-linear. As an example, taking fruit consumption in 55-65 year old men and CHD mortality; 0.94 relative risk of CHD mortality per 100g increase fruit consumption is mathematically: log(0.94) / 100g = -0.059 per 100g increase fruits consumption. Then for a certain increase in fruits consumption (21.2g as observed in 30% price reduction of F&V in this age group), we calculated the reduction in CHD mortality as: 1-exp (0.059 per 100g increased consumption) = e.g., 1-exp(0.059 x (21.2/100)) = 0.013 (this is a 1.3% reduction). This reduction is then applied to the baseline number of deaths in each year 2015-2030. These are then summed. This is then repeated for each age and gender

**References.**

1. Afshin A, Penalvo JL, Del Gobbo L, Silva J, Michaelson M, O'Flaherty M, et al. The prospective impact of food pricing on improving dietary consumption: A systematic review and meta-analysis. PLoS One. 2017;12(3):e0172277. Epub 2017/03/02. doi: 10.1371/journal.pone.0172277. PubMed PMID: 28249003; PubMed Central PMCID: PMCPMC5332034
2. Green R, Cornelsen L, Dangour AD, Turner R, Shankar B, Mazzocchi M, et al. The effect of rising food prices on food consumption: systematic review with meta-regression. Bmj. 2013;346:f3703. Epub 2013/06/19. doi: 10.1136/bmj.f3703. PubMed PMID: 23775799; PubMed Central PMCID: PMCPMC3685509
3. Penalvo JA, Ashkan. Mozaffarian, Dariush. Personal communication – March 2016
4. Afshin A, Penalvo J, Del Gobbo L, Kashaf M, Micha R, Morrish K, et al. CVD Prevention Through Policy: a Review of Mass Media, Food/Menu Labeling, Taxation/Subsidies, Built Environment, School Procurement, Worksite Wellness, and Marketing Standards to Improve Diet. Curr Cardiol Rep. 2015;17(11):98. doi: 10.1007/s11886-015-0658-9. PubMed PMID: 26370554; PubMed Central PMCID: PMCPMC4569662.
5. Potter J, Finnegan J, Guinard J, Huerta E, Kelder S, Kristal A, et al. National Health Institute, National Cancer Institute. 5 a day for better health program evaluation report 2000: National Health Institute, National Cancer Institute. <http://www.scgcorp.com/docs/5_a_Day_Booklet_sm.pdf - accessed December 2015>
6. Micha R, Penalvo JL, Cudhea F, Imamura F, Rehm CD, Mozaffarian D. Association Between Dietary Factors and Mortality From Heart Disease, Stroke, and Type 2 Diabetes in the United States. Jama. 2017;317(9):912-24. Epub 2017/03/08. doi: 10.1001/jama.2017.0947. PubMed PMID: 28267855
