# Supplementary figures and images for: Reducing US cardiovascular disease burden and disparities through national and targeted dietary policies: A modelling study
Source: PLoS Med. 2017 Jun 6;14(6):e1002311. doi: 10.1371/journal.pmed.1002311 (PMC5460790; doi:10.1371/journal.pmed.1002311)

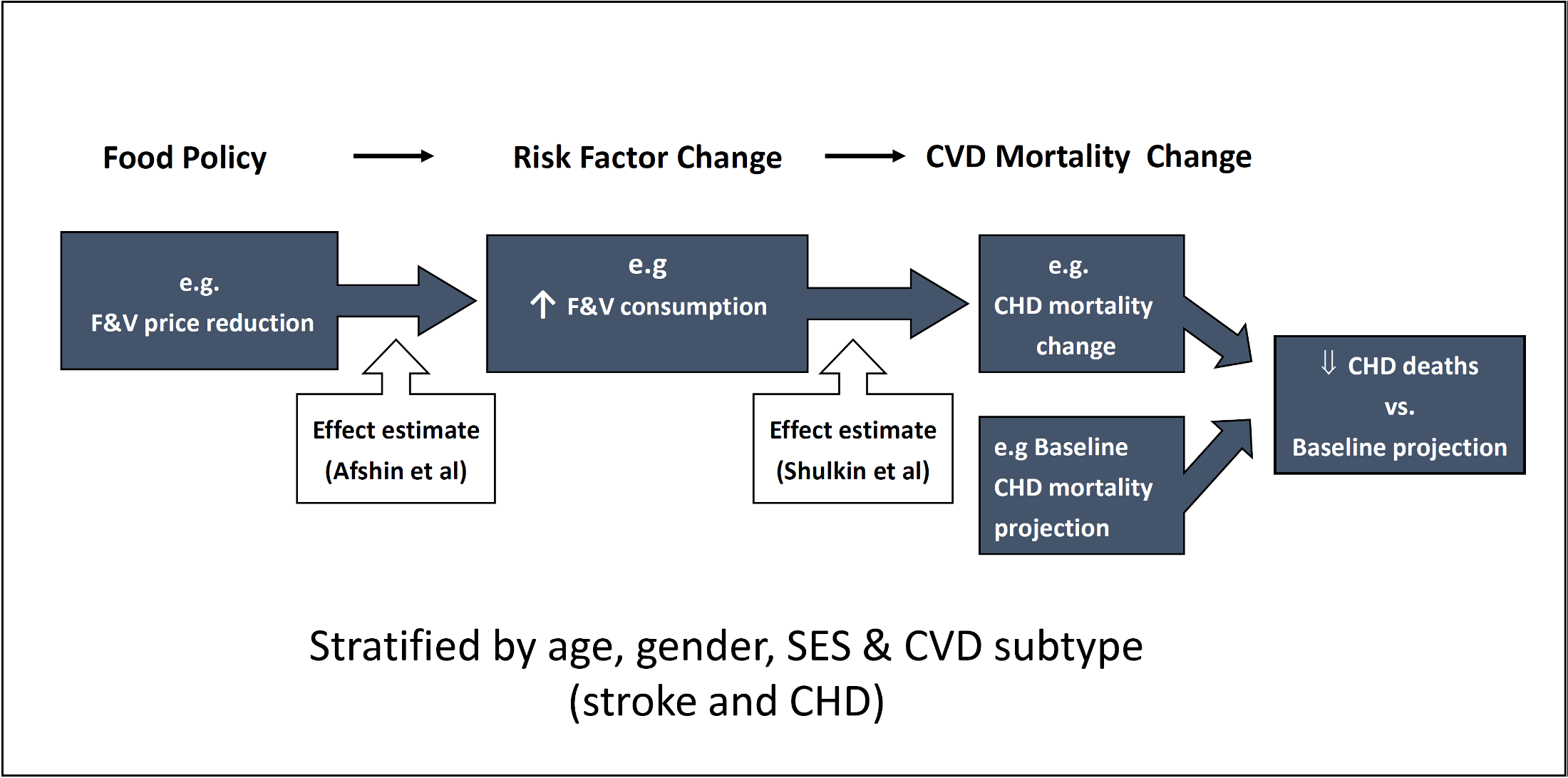

Supplement: S1 Fig — (TIFF) [file pmed.1002311.s002.tiff]
