# Supplementary material for: Reducing US cardiovascular disease burden and disparities through national and targeted dietary policies: A modelling study
Source: PLoS Med. 2017 Jun 6;14(6):e1002311. doi: 10.1371/journal.pmed.1002311 (PMC5460790; doi:10.1371/journal.pmed.1002311)
Supplement: S1 Table — (DOC) [file pmed.1002311.s004.doc]

**S1 Table**

**Mass Media Campaign Coverage**

Table below outlines the coverage estimates by age and gender of the nationwide mass media campaign modelled in our study. Here we used the full effect size 7% (4-9%) for the age and gender groups with the largest effect size measured in the ‘Five a day campaign’ and apportioned reduced effect size to each other sub-group according to proportional lower observed effect size. These coverage estimates were applied to the regression coefficient for each age, gender and race group derived from the media campaign effect size upon F&V consumption coupled with effect size of change in consumption upon CHD and stroke mortality. The data in the table below was derived from coverage data from evaluation of the US ‘5 a day’ campaign[1].

**Coverage estimates of mass media campaign stratified by age and gender.**

| **Age** | **Men** | **Women** |
| --- | --- | --- |
| **25-34** | 1.000 | 0.831 |
| **35-44** | 0.875 | 0.727 |
| **45-54** | 0.653 | 0.543 |
| **55-64** | 0.431 | 0.358 |
| **65-74** | 0.897 | 0.746 |
| **75-84** | 0.897 | 0.746 |
| **85+** | 0.897 | 0.746 |

1. Potter J, Finnegan J, Guinard J, Huerta E, Kelder S, Kristal A, et al. National Health Institute, National Cancer Institute. 5 a day for better health program evaluation report 2000: National Health Institute, National Cancer Institute.
