# Supplementary material for: Reducing US cardiovascular disease burden and disparities through national and targeted dietary policies: A modelling study
Source: PLoS Med. 2017 Jun 6;14(6):e1002311. doi: 10.1371/journal.pmed.1002311 (PMC5460790; doi:10.1371/journal.pmed.1002311)
Supplement: S2 Table — (DOC) [file pmed.1002311.s005.doc]

**S2 Table**

**Expected deaths (baseline deaths) and cumulative Deaths Prevented or Postponed (DPP) and CVD mortality reduction (%) from 2015-2030 achieved through all policies modelled.** *Stratified by age. and CVD subtype. (95% uncertainty intervals)*

|  | | **Age** | | | | | | |
| --- | --- | --- | --- | --- | --- | --- | --- | --- |
|  |  | **25-34** | **35-44** | **45-54** | **55-64** | **65-74** | **75-84** | **85+** |
| **Media campaign** | **Baseline deaths** | 26,500 (26,000-27,000) | 119,300 (116,600-122,100) | 339,100 (331,200-347,200) | 784,500 (767,500-801,500) | 1,372,200 (1,341,600-1,404,500) | 1,951,400 (1,910,300-1,994,000) | 2,469,800 (2,419,200-2,521,500) |
|  | **DPP** | 400 (300-400) | 1,300 (1,100-1,600) | 2,300 (1,900-2,800) | 2,700 (2,300-3,300) | 6,900 (5,900-8,500) | 5,200 (4,500-6,300) | 6,900 (6,000-8,400) |
|  | **Mortality reduction** | 1.4% | 1.1% | 0.7% | 0.3% | 0.5% | 0.3% | 0.3% |
| **10% SSB tax** | **Baseline deaths** | 26,500 (25,900-27,000) | 119,300 (116,600-122,100) | 339,100 (331,000-347,300) | 784,500 (767,400-802,300) | 1,372,200 (1,341,000-1,404,700) | 1,951,400 (1,909,000-1,994,500) | 2,469,800 (2,420,200-2,521,500) |
|  | **DPP** | 600 (500-700) | 2,800 (2,200-3,400) | 5,200 (3,900-6,600) | 7,300 (5,400-9,500) | 6,500 (4,400-9,200) | 3,900 (2,500-5,700) | 4,800 (3,200-6,900) |
|  | **Mortality reduction** | 2.3% | 2.3% | 1.5% | 0.9% | 0.5% | 0.2% | 0.2% |
| **10% FV subsidy** | **Baseline deaths** | 26,500 (26,000-27,100) | 119,300 (116,500-122,200) | 339,100 (331,200-347,300) | 784,500 (767,400-802,000) | 1,372,200 (1,341,300-1,404,500) | 1,951,400 (1,909,400-1,994,100) | 2,469,800 (2,418,600-2,521,300) |
|  | **DPP** | 1,100 (1,000-1,300) | 4,200 (3,700-4,800) | 11,300 (9,800-12,700) | 24,400 (21,500-27,200) | 37,600 (32,900-42,300) | 31,400 (27,600-35,300) | 40,400 (35,500-45,400) |
|  | **Mortality reduction** | 4.2% | 3.5% | 3.3% | 3.1% | 2.7% | 1.6% | 1.6% |
| **SNAP 30% FV subsidy** | **Baseline deaths** | 26,500 (25,900-27,100) | 119,300 (116,500-122,200) | 339,100 (331,100-347,400) | 784,500 (767,700-801,800) | 1,372,200 (1,341,400-1,403,800) | 1,951,400 (1,909,900-1,992,600) | 2,469,800 (2,420,000-2,520,900) |
|  | **DPP** | 300 (300-400) | 1,900 (1,500-2,200) | 5,200 (4,200-6,100) | 7,300 (5,800-8,900) | 9,700 (8,000-11,300) | 4,500 (3,700-5,300) | 6,200 (5,000-7,400) |
|  | **Mortality reduction** | 1.3% | 1.6% | 1.5% | 0.9% | 0.7% | 0.2% | 0.2% |
| **Combined** | **Baseline deaths** | 26,500 (26,000-27,000) | 119,300 (116,500-122,100) | 339,100 (331,400-347,200) | 784,500 (767,700-801,300) | 1,372,200 (1,341,000-1,403,800) | 1,951,400 (1,909,700-1,994,700) | 2,469,800 (2,419,400-2,522,300) |
|  | **DPP** | 2,200 (2,000-2,400) | 9,000 (7,900-9,900) | 21,200 (18,700-23,400) | 40,700 (36,500-44,300) | 54,100 (48,100-59,700) | 43,800 (39,200-48,200) | 57,000 (51,000-62,900) |
|  | **Mortality reduction** | 8.3% | 7.5% | 6.3% | 5.2% | 3.9% | 2.2% | 2.3% |
