# Supplementary material for: Reducing US cardiovascular disease burden and disparities through national and targeted dietary policies: A modelling study
Source: PLoS Med. 2017 Jun 6;14(6):e1002311. doi: 10.1371/journal.pmed.1002311 (PMC5460790; doi:10.1371/journal.pmed.1002311)
Supplement: S3 Table — (DOC) [file pmed.1002311.s006.doc]

**S3 Table**

**Probabilistic sensitivity analysis in the US IMPACT Food Policy model** parameters.

| **Input parameters** | **Parameter description** | ***Type of distribution and functions*** | **Source** |
| --- | --- | --- | --- |
| **Population and deaths** | | | |
| Population counts. | Number of people in each age and gender group each year. | No uncertainty assumed for population forecast | Population – US Bureau 2012 National Population Projections[1, 2]. |
| CHD and stroke mortality stratified by age and sex. | Number of deaths due to CHD or stroke by age and gender each year. | Log normal distribution +/- 20% | Modelling future cardiovascular disease mortality in the united states: National trends and racial and ethnic disparities – Pearson-Stuttard et al[3] |
| Stroke deaths stratified by age and sex.  Ischaemic: Haemorrhagic ratio | Ratio of ischaemic: haemogghaic stroke deaths by age and sex each year. | Log normal distribution | Modelling future cardiovascular disease mortality in the united states: National trends and racial and ethnic disparities – Pearson-Stuttard et al[3]  Heart Disease and Stroke Statistics-2016 Update: A Report From the American Heart Association. [4] |
| **Estimation of DPP** | | | |
| Regression coefficient of change in F&V intake upon CHD and stroke mortality | Effect estimate of change in fruits and vegetable (individually) intake upon CHD and stroke mortality | Log normal distribution | Micha et al. Major dietary risk factors for cardiometabolic disease: current evidence for causal effects and effect sizes[5].  For stroke used value of regression coefficient weighted for prevalence of ischemic and hemorrhagic stroke |
| Regression coefficient of change in SSB intake upon CHD and stroke mortality | Effect estimate of change in fruits and vegetable (individually) intake upon CHD and stroke mortality | Log normal distribution |  |
| **Fruit, Vegetables and SSB** | | | |
| Intake and projections | Intake of fruits, vegetables and SSBs in baseline year and to 2030. | Log normal | Intake - National health and nutrition examination survey (NHANES), 2009-2012[6]  Projections – Adapted from National health and nutrition examination survey. |
| F&V Price reduction | Change in fruits and vegetables intake associated with reduction in price. | Pert, Best estimate 14%, CIs 11-17% | The effect of food pricing on dietary behaviours and adiposity: a systematic review and meta-analysis. Afshin et al[7] |
| SSB Price increase | Change in sugar sweetened beverage intake associated with increase in price. | Pert, Best estimate 7%, CIs 4-10% | The effect of food pricing on dietary behaviours and adiposity: a systematic review and meta-analysis. Afshin et al[7] |
| Mass Media Campaign | Change in fruits, vegetables and sugar sweetened beverage intake associated with mass media campaign. | Pert, +/-20% | Afshin A, Ajala ON, Nguyen AB et al. Effectiveness of Mass Media Campaigns for Improving Dietary Behaviours: A Systematic Review and Meta-analysis. Afsin et al[8, 9] |
| MMC Policy Coverage | Variation in efficacy of mass media campaign by age and gender | Pert, +/-20% | Potter J et al. 5 a day for better health program evaluation:  National health institute, national cancer institute. 2000[10] |
| Decaying impact of MMC | Attrition of effectiveness (change in dietary intake) of mass media campaign over time. | Pert, best estimate (20% after 5 years), LL5%, UL 40% | Assumption |

**References.**

1. US Census Bureau Projections; <http://www.census.gov/population/projections/data/national/2012/summarytables.html> - accessed 21st December 2015
2. US Census Bureau. US demography by race. http://www.census.gov/topics/population/race.html. 2005. - accessed 22^nd^ May 2015
3. Pearson-Stuttard J, Guzman-Castillo M, Penalvo JL, Rehm CD, Afshin A, Danaei G, et al. Modeling Future Cardiovascular Disease Mortality in the United States: National Trends and Racial and Ethnic Disparities. Circulation. 2016;133(10):967-78. doi: 10.1161/CIRCULATIONAHA.115.019904. PubMed PMID: 26846769; PubMed Central PMCID: PMCPMC4783256.
4. Mozaffarian D, Benjamin EJ, Go AS, Arnett DK, Blaha MJ, Cushman M, et al. Heart Disease and Stroke Statistics-2016 Update: A Report From the American Heart Association. Circulation. 2016;133(4):e38-e360. doi: 10.1161/CIR.0000000000000350. PubMed PMID: 26673558
5. Micha R, Penalvo JL, Cudhea F, Imamura F, Rehm CD, Mozaffarian D. Association Between Dietary Factors and Mortality From Heart Disease, Stroke, and Type 2 Diabetes in the United States. Jama. 2017;317(9):912-24. Epub 2017/03/08. doi: 10.1001/jama.2017.0947. PubMed PMID: 28267855
6. National Health and Nutrition Examination Survey (NHANES), 2009-2012. http://wwwn.cdc.gov/nchs/nhanes/search/datapage.aspx?Component=dietary&CycleBeginYear=2009 - accessed May 2015
7. Afshin A, Penalvo JL, Del Gobbo L, Silva J, Michaelson M, O'Flaherty M, et al. The prospective impact of food pricing on improving dietary consumption: A systematic review and meta-analysis. PLoS One. 2017;12(3):e0172277. Epub 2017/03/02. doi: 10.1371/journal.pone.0172277. PubMed PMID: 28249003; PubMed Central PMCID: PMCPMC5332034
8. Afshin A, Penalvo J, Del Gobbo L, Kashaf M, Micha R, Morrish K, et al. CVD Prevention Through Policy: a Review of Mass Media, Food/Menu Labeling, Taxation/Subsidies, Built Environment, School Procurement, Worksite Wellness, and Marketing Standards to Improve Diet. Curr Cardiol Rep. 2015;17(11):98. doi: 10.1007/s11886-015-0658-9. PubMed PMID: 26370554; PubMed Central PMCID: PMCPMC4569662
9. Afshin A, Ajala ON, Nguyen AB, Mozaffarian D. Effectiveness of Mass Media Campaigns for Improving Dietary Behaviors: A Systematic Review and Meta-analysis. Circulation 2013; 127: AP087

.

1. Potter J, Finnegan J, Guinard J, Huerta E, Kelder S, Kristal A, et al. National Health Institute, National Cancer Institute. 5 a day for better health program evaluation report 2000: National Health Institute, National Cancer Institute. <http://www.scgcorp.com/docs/5_a_Day_Booklet_sm.pdf - accessed December 2015>
