# Supplementary material for: Reducing US cardiovascular disease burden and disparities through national and targeted dietary policies: A modelling study
Source: PLoS Med. 2017 Jun 6;14(6):e1002311. doi: 10.1371/journal.pmed.1002311 (PMC5460790; doi:10.1371/journal.pmed.1002311)
Supplement: S4 Table — (DOC) [file pmed.1002311.s007.doc]

**S4 Table**

**Key assumptions for the US IMPACT Food Policy Model**

| **Assumption** | **Rationale** | **Assumption risk** |
| --- | --- | --- |
| Trends in CHD and stroke mortality rates from 1980 – 2012 will continue from 2015-2030. | Validated CVD mortality projections to 2030 (Pearson-Stuttard et al[1] | Assumes no ‘black swan’ i.e. unpredictable events to occur from 2015-2030 that changes mortality trends substantially. |
| Efficacy of price interventions |  |  |
| Efficacy of mass media campaign varies by age and gender. | Used empirical evidence from ‘5 a-day’ US mass media campaign evaluation[2] | If mass media campaign is equally as effective in all age and gender sub-groups, our model will under estimate the effectiveness (DPPs) of the policy scenario. |
| Effectiveness of mass media campaign to reduce over time after the campaign concludes. We model full effectiveness in year 1 reducing to 20% effectiveness in year 5 with linear reduction from year 1 to 5. We then model sustained effectiveness of 20% for the remaining 10 years. | Unlike price change interventions, media campaigns (e.g. 5 a-day) are usually fixed term. We therefore assume it is effective when ongoing (year 1) after which the effectiveness reduces. | If the effect of the media campaign was sustained at full effectiveness throughout the 14 years following the campaign ending, we will have conservatively underestimated the effect (DPPs) generated by this policy over the period 2015-30. |

**References.**

1. Pearson-Stuttard J, Guzman-Castillo M, Penalvo JL, Rehm CD, Afshin A, Danaei G, et al. Modeling Future Cardiovascular Disease Mortality in the United States: National Trends and Racial and Ethnic Disparities. Circulation. 2016;133(10):967-78. doi: 10.1161/CIRCULATIONAHA.115.019904. PubMed PMID: 26846769; PubMed Central PMCID: PMCPMC4783256.
2. Potter J, Finnegan J, Guinard J, Huerta E, Kelder S, Kristal A, et al. National Health Institute, National Cancer Institute. 5 a day for better health program evaluation report 2000: National Health Institute, National Cancer Institute. <http://www.scgcorp.com/docs/5_a_Day_Booklet_sm.pdf - accessed December 2015>
